# The Realization of Scalar Inferences: Context Sensitivity without Processing Cost

**DOI:** 10.1371/journal.pone.0063943

**Published:** 2013-05-16

**Authors:** Stephen Politzer-Ahles, Robert Fiorentino

**Affiliations:** Department of Linguistics, University of Kansas, Lawrence, Kansas, United States of America; CSIC-Univ Miguel Hernandez, Spain

## Abstract

*Scalar inference* is the phenomenon whereby the use of a less informative term (e.g., *some of*) is inferred to mean the negation of a more informative term (e.g., to mean *not all of*). Default processing accounts assume that the interpretation of *some of* as meaning *not all of* is realized easily and automatically (regardless of context), whereas context-driven processing accounts assume that it is realized effortfully and only in certain contexts. In the present study, participants’ self-paced reading times were recorded as they read vignettes in which the context did or did not bias the participants to make a scalar inference (to interpret *some of* as meaning *not all of*). The reading times suggested that the realization of the inference was influenced by the context, but did not provide evidence for processing cost at the time the inference is realized, contrary to the predictions of context-driven processing accounts. The results raise the question of why inferencing occurs only in certain contexts if it does not involve extra processing effort.

## Introduction

Language comprehension involves rapid integration of various types of meaning, including coming from words and phrases themselves (i.e., semantic information) and that coming from extra-linguistic inferences about what a speaker must intend to express with an utterance (i.e., pragmatic information). Although this sort of integration is ubiquitous in natural language use, the dynamics of semantic and pragmatic meaning computation are not well understood. Particularly, there is ongoing debate regarding whether speakers compose inferential pragmatic aspects of meaning immediately and effortlessly, or whether they first compose the semantic meaning and then integrate pragmatic information at a later processing stage.

One test case for investigating this question is *scalar inference*, the phenomenon whereby the use of a weaker term (e.g., *some of*) is inferred to mean the negation of a stronger term (e.g., to mean *not all of*). Consider the exchange in (1):

1
  - Are all of the students in your department hardworking?
  - Some of them are.

(1b) is often understood as meaning “No, not all of them are.” This interpretation, however, is not part of the inherent semantics of the quantifier *some of*. Rather, “not all” is thought to be a meaning that is generated through a pragmatic enrichment process [1]: a hearer expects that a cooperative speaker will use the most informative term possible, and thus the speaker’s choice not to use the stronger term *all of* must mean that the stronger term is not true–in other words, that *some of* means “not all of” [1], [2]. On the other hand, the inherent, semantic meaning of *some of* is “at least one”, and could be consistent with *all of*. The fact that the pragmatic interpretation (“not all”) is an enriched meaning and not part of the basic semantic meaning (“at least one”) is evident from the fact that the pragmatic interpretation can be cancelled (e.g., in 2a) without resulting in a nonsensical sentence [3], whereas the semantic meaning cannot (e.g., 2b):

2
  - Some of the students in this department are hardworking. In fact, *all* of them are.
  - Some of the students in this department are hardworking. #In fact, *none* of them are.

There are competing psycholinguistic accounts regarding how the pragmatic meanings of scalar terms like *some of* are realized online (for reviews, see [4], [5]). Broadly speaking, *context-driven models* [1] assume that realizing a scalar inference requires extra effort (inferring the speaker’s intentions), whereas *default models* [6] hold that the inference-based pragmatic meaning is realized effortlessly and automatically. Context-driven models predict that, because the parser avoids exerting extra effort, scalar inferences are only realized in contexts where they are relevant, and thus the inference does not occur until after the context and the semantic meaning of an utterance have been processed. Default models, on the other hand, predict that inferencing is context-independent and occurs immediately.

We note that there is not universal agreement on whether the inference-based meaning of some of is pragmatic, as claimed above; a grammatical account of scalar inference [7] argues that the “not all” meaning is realized through the insertion of an operator at the logical form of the sentence and thus is still semantics-based. Under such an account, scalar inference is not a case of pragmatic processing, but the questions raised above (specifically, the default or context-based nature of the process subserving the realization of the enriched meaning of some of) are still relevant regardless of whether the locus of such an operation is pragmatic or semantic.

Most psycholinguistic studies testing these models have focused on the speed at which scalar inferences occur (see, e.g., [4], [8]–[10]). Little is known, however, about whether inferences entail a processing cost. More direct empirical evidence for or against processing costs in scalar inferencing is necessary if we are to understand how meaning is realized during online comprehension. As the predictions of the default and context-driven processing models rely upon their assumptions about whether inferencing is effortful, data regarding the speed of inferencing is not sufficient to adjudicate between these models; data regarding the costs of inferencing are also needed. In short, our understanding of the psychology of inferencing cannot be complete without an account of the processing costs involved.

There is some evidence that the availability of processing resources influences comprehenders’ offline judgments of sentences with scalar terms [11], [12]. Studies using speeded verification have shown that comprehenders often take longer to evaluate inference-based readings of scalars than semantic readings [13], [14], [10] (but see [15]). The results of some of these studies are difficult to draw conclusions from, however, given that the responses could have been influenced both by time to realize the inference and time to verify or reject the different meanings [4]. Few studies have attempted to directly measure processing costs elicited by scalar terms during online comprehension. Such a study would involve presenting comprehenders with scalar terms in contexts where the inference is made and those where it is not, and comparing their processing of these terms using some measure that is sensitive to the occurrence of greater processing effort, such as reading times [16]. Breheny, Katsos, and Williams ([17]; hereafter “BKW”) performed such an experiment: they compared reading times to the Greek equivalent of *some of* in contexts that bias readers towards making the implicature (“upper-bound” contexts, where what is relevant to the discourse is whether *not all* is true, and thus *some of* is likely to be interpreted as *not all of*) and in contexts that do not (“lower-bound” contexts, where what is relevant is whether *any* is true, and thus *some of* is unlikely to be interpreted as *not all*); see the examples in (3).

3
  - **Upper-bound**: Mary asked John whether he intended to host all of his relatives in his tiny apartment. John replied that he intended to host **some of his relatives**. The rest would stay in a nearby hotel.
  - **Lower-bound**: Mary was surprised to see John cleaning his apartment and she asked the reason why. John told her that he intended to host **some of his relatives**. The rest would stay in a nearby hotel.

BKW found slower reading times to *some of* in the upper-bound (inference-supporting) context, and interpreted that as evidence that realizing the inference involves extra processing effort. Their contexts, however, differed in more ways than boundedness–in particular, the phrase “his relatives” in the critical region is repeated in the upper-bound context (and thus is infelicitous, since a pronoun would be expected), whereas it is new in the lower-bound context (see similar arguments in [4]). Thus, it is questionable whether the reading time slowdown observed by BKW is evidence for additional processing involved in realizing an inference, or is due to unrelated factors.

The present study tests whether the realization of a scalar inference triggers processing costs, by adopting the design of BKW but using maximally similar upper- and lower-bound contexts. In this study, the only difference between the contexts is whether the context sentence uses the quantifier *all* (as in the upper-bound example in (3)) or *any*. Including *all* in the context makes the upper bound relevant in the discourse and thus encourages the comprehender to interpret *some of* as *not all of*, whereas *any* makes the upper bound irrelevant and discourages the inference. Furthermore, to verify whether the inference is ultimately realized, we follow BKW in including a sentence with “the rest” after the critical sentence with *some of*. If the reader has interpreted *some of* as meaning *not all of*, then she is aware of a remaining set of referents (e.g. “relatives”) and thus more easily able to link “the rest” with a referent. Therefore, faster reading times at “the rest” in the upper-bound than the lower-bound context indicate that the inference has been realized in the upper-bound but not the lower-bound context. “The rest” also provides a secondary test of the speed of inferencing. Hartshorne and Snedeker (unpublished data; hereafter H&S), examining another kind of context manipulation, found faster reading times at “the rest” in the inference-supporting context when “the rest” appeared about 2500 ms after the quantifier but not when it appeared about 900 ms after; the authors took this as evidence that the inference takes over 900 ms to realize.

In the current study, we provide evidence that realizing scalar inferences does not trigger a processing cost, as reading times to the quantifier did not differ between contexts that encourage the reader to make the inference and contexts that do not, even though the inference was eventually realized in the inference-encouraging context and not the inference-discouraging context (as evidenced by reading times at “the rest”).

## Methods

### Ethics Statement

The procedures used in this study were approved by the Human Subjects Committee of Lawrence (#20261).

### Participants

Twenty-eight native English speakers from the University of Kansas (20 women; ages 18–56, median 19) participated in the study for payment. Participants provided their written informed consent.

### Materials

Forty-eight sets of four-sentence vignettes were constructed following the template in (4); slashes indicate how the vignettes were divided into segments for the self-paced reading task (see Procedure). A full list of materials is available as File S1 in the online supplementary materials for this article.

4
  - **Upper-bound** ***some*** **:** Mary was preparing to throw a party for John’s relatives./She asked John whether *all* of them were staying in his apartment./John said that/some of them */*were./He added/that/the rest */*would be/staying/in a hotel.
  - **Lower-bound** ***some*** **:** Mary was preparing to throw a party for John’s relatives./She asked John whether *any* of them were staying in his apartment./John said that/some of them */*were./He added/that/the rest */*would be/staying/in a hotel.
  - **Upper-bound** ***only some*** **:** Mary was preparing to throw a party for John’s relatives./She asked John whether *all* of them were staying in his apartment./John said that/only some of them */*were./He added/that/the rest */*would be/staying/in a hotel.
  - **Lower-bound** ***only some*** **:** Mary was preparing to throw a party for John’s relatives./She asked John whether *any* of them were staying in his apartment./John said that/only some of them */*were./He added/that/the rest */*would be/staying/in a hotel.

In each set, the first sentence establishes a set of items or people (e.g., John’s relatives). The second sentence establishes an upper- or lower-bound context by asking about either *all of* them or *any of* them. The third sentence includes a response to the previous indirect question, using *some of*, which is predicted to be interpreted as *not all* in the upper-bound (since “all” is relevant in that context, but was not used) but not the lower-bound context (since “all” is not relevant in that context). Finally, the fourth sentence includes a mention of *the rest* of the set. “The rest” was always followed by “would be” and two or three more segments of one or more words each. The only difference between contexts is the use of *all* or *any* in the second sentence.

In addition to manipulating the boundedness of the context, we also manipulated the quantificational expression in the third sentence. Each of the vignette types above also has a counterpart written using *only some of* rather than *some of* (see (4)); serving to make the *not all* interpretation semantically explicit. This is important because comparing reading times between sentences in which the quantifier was interpreted pragmatically and those in which the quantifier was interpreted semantically involves comparing across sentences with different meanings, which may take different amounts of time or effort to interpret or verify (see [10]). A control comparison is needed to isolate these other factors from pragmatic inferencing. If a difference between upper- and lower-bound conditions is due to pragmatic inferencing rather than other factors, then that difference should appear in the implicit upper-bound (*some of*) sentences but not in the explicit upper-bound (*only some of*) sentences.

In addition to the critical stimuli, 144 filler vignettes were created. Forty-eight follow the same format as the critical sentences but do not include *the rest*; this is both to make sure participants cannot predict *the rest* in every item and to make sure that *some of* is not always associated with *the rest* (which is an explicit cue to the inference). Forty-eight use *all of* rather than *some of* or *only some of* in the third sentence, to make sure participants cannot predict *some of* or *only some of* in every item; these items also do not include *the rest*. The last 48 use various other quantifiers in the third sentence (*many of*, *most of*, *several of*, *a few of*, *none of*, and numbers) to increase the variety of lexical alternatives to *some of* present in the experimental context, which has been shown to influence the speed and outcome of scalar inferencing [9].

### Procedure

Participants read the vignettes in a non-cumulative moving-window self-paced reading paradigm [16], administered using the Presentation software package (Neurobehavioral Systems, Inc.). In each trial, the passage was shown on the screen with all the characters replaced with dashes; the participant pressed a button on a gamepad to show a phrase (at which point the dashes were replaced with the phrase). With each button press, the currently displayed phrase turned back into dashes and the next phrase was displayed. Participants were instructed to read the sentences for comprehension at a natural reading speed. One-third of the sentences were followed by comprehension questions, e.g. “Who was Mary throwing a party for?” The comprehension questions never targeted aspects of the passage that depend upon the interpretation of quantifiers. The main experiment was preceded by eight practice items. The procedure took 40–50 minutes to complete, with five breaks.

### Data Analysis

Reading times for filler items and for the first two segments of the critical items (the context segments which were presented as entire sentences) were excluded from all analyses. The remaining reading times were log-transformed for normality, and outliers for each participant and item removed based on visual inspection. (This method of outlier trimming is recommended by [18]. The pattern of results reported below was also observed using other outlier-trimming methods such as a flat criterion as in H&S, a subject-wise standard deviation criterion, and a hybrid method based on that described in BKW–first removing observations below 150 ms or greater than 3 times the overall mean of observations in a given region, then removing observations that differ by more than three standard deviations from that subject’s mean for that region.) Linear mixed models with random intercepts for participants and items [19] were fit with predictors Quantifier (*some*, *only some*), Boundedness (*upper, lower*), and sentence Region, and model comparison was conducted with log-likelihood tests. Accuracy was analyzed using generalized linear mixed models with predictors Quantifier and Boundedness. Evaluation of the significance of model coefficients was conducted using Markov Chain Monte Carlo sampling. All data are available in File S2 in the online version of this article.

## Results

### Accuracy

Participants responded correctly to 93.8% of items in the upper-bound *some* condition, 90.2% in lower-bound *some*, 94.6% in upper-bound *only some*, and 91.1% in lower-bound *only some*. There were no significant differences in accuracy across conditions and no interaction (*χ* ^2^s<2.3, *p*s>.130).

### Reading Times

**Figure 1:**
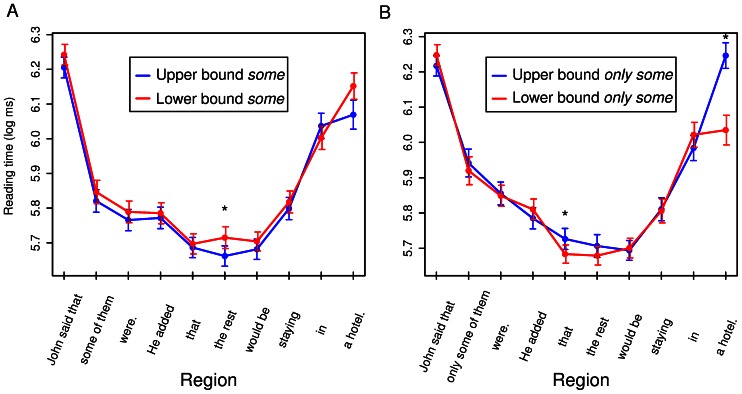
Segment-by-segment reading times. Reading times by region for the last two sentences in *some* vignettes (panel A) and *only some* vignettes (panel B). Regions showing a significant effect of boundedness for a given quantifier type are indicated with an asterisk. Error bars represent ±.5 standard errors of the mean.

Figure 1 shows the reading times for the last two sentences of the vignettes. It is evident that, for *some* sentences, “the rest” was read more slowly in the lower-bounded context, whereas such a pattern was not observed in *only some* sentences. It is also apparent that there is no slowdown at the quantifier in *some* sentences in the upper-bound context. Statistical analysis confirmed these observations.

After outlier removal (see Data analysis), 12,105 observations remained for analysis. Standard deviations for the random effects in the saturated model were as follows: Items: 0.038; Participants: 0.167; Residual: 0.282. There was a significant three-way interaction between Region, Quantifier, and Boundedness (*χ* ^2^(9) = 23.10, *p* = .006). For *some* sentences, reading times for “the rest” were significantly slower in the lower-bound than upper-bound context (*b* = 0.054, SE = 0.022, *t* = 2.42, *p* = .014, 95% CI: 0.01–0.097). No significant difference was observed at “the rest” in *only some* sentences, and the trend was in the opposite direction (*b* = −.030, SE = 0.022, *t* = −1.37, *p* = .167, 95% CI: −0.074–0.013). The only regions where *only some* sentences showed a boundedness effect were “that” (the region preceding “the rest”; *b* = −0.046, SE = 0.022, *t* = −2.09, *p* = .038, 95% CI: −0.09– −0.003) and the sentence-final region (*b* = −0.185, SE = 0.055, *t* = −23.36, *p*<.001, 95% CI: −0.293– −0.077). No significant effect of context was observed at the quantifier (“some of” or “only some of”) or the following two regions, either for *some* sentences (*b*s<0.032, SEs>0.022, *t*s<1.38, *p*s>.291) or for *only some* sentences (|*b*|s<0.021, SEs>0.022, |*t*|s<0.96, *p*s>.329). The 95% confidence intervals for these null effects were as follows: *some* sentences: −0.02–0.067 (quantifier), −0.023–0.064 (quantifier+1), −0.036–0.05 (quantifier+2); *only some* sentences: −1.606–1.57 (quantifier), −0.835–0.816 (quantifier+1), −1.857–1.9 (quantifier+2).

Based on the confidence intervals of the effects, the fact that significant effects were observed in other predicted places (i.e., at “the rest” in the *some* sentences), and the fact that the numerical effect of context at the quantifier position was not even in the same direction as in BKW, it is not likely that the null effect of context at the quantifier region could have been due to lack of power. Nevertheless, we performed a simulation-based post-hoc power analysis to test whether the design had sufficient power to detect an effect of similar size if such an effect existed (although the usefulness of such post-hoc analyses has been challenged [20], [21]). The analysis revealed an observed power of.981, suggesting that with the number of participants and items tested we had a 98.1% chance to detect an effect of the size that BKW reported. Details of this analysis are in Appendix S1.

**Figure 2:**
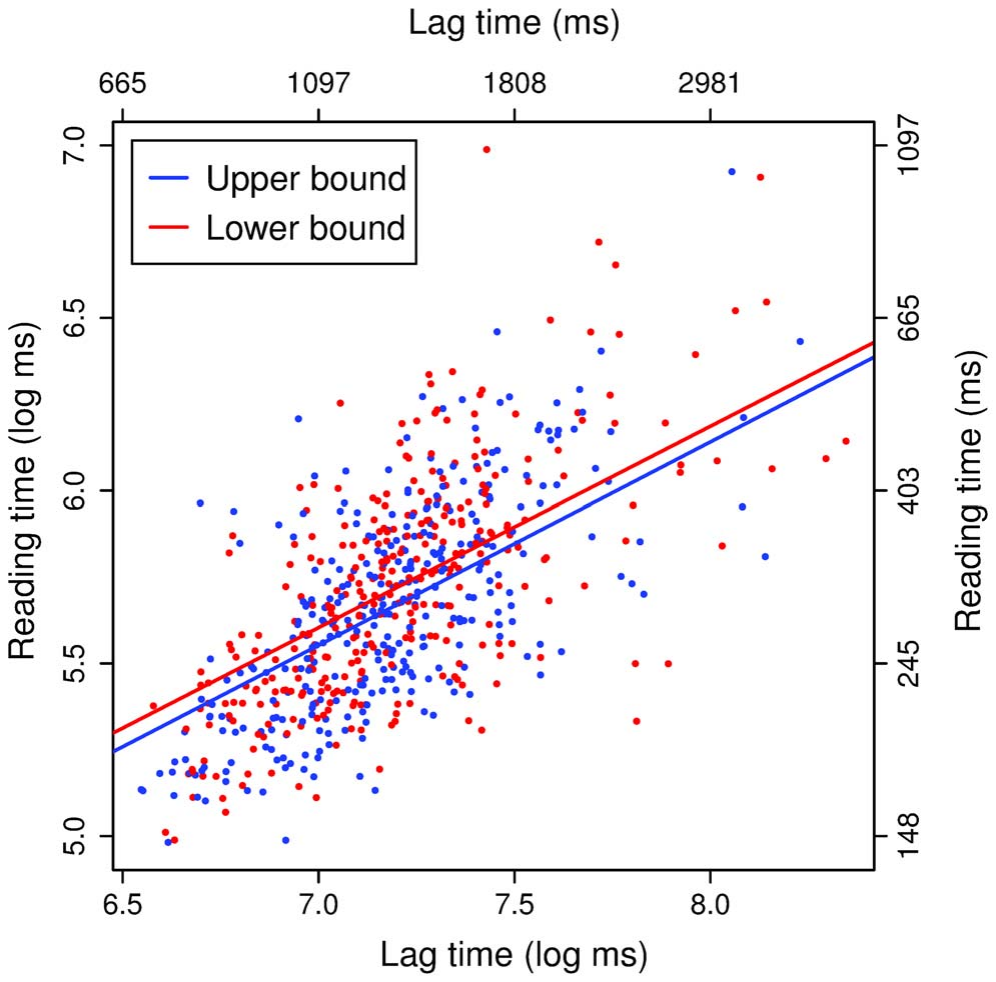
Reading time vs. lag time. Relationship between reading times on “the rest” and lag between the quantifier and “the rest” for upper-bound (dots and solid line) and lower-bound (triangle and dashed line) contexts. Points represent individual observations, and regression lines represent predictions from a mixed model with fixed effects of Boundedness, Lag Time, and their [non-significant] interaction. The bottom and left axes show log lag time and log reading time respectively, and the top and right axes show raw lag time and raw reading time.

Because H&S found an effect of context when “the rest” appeared about 2500 ms after the quantifier but not when it appeared about 900 ms after, we also calculated the lag between quantifier and “the rest” in the implicit upper-bound (*some of*) vignettes. The average lag was 1435 ms. A mixed model on the reading times at “the rest” and the following region, for the *some* sentences only, showed that the effect of context did not interact with the lag time (*χ* ^2^(2) = 0.48, *p* = .788), thus not providing evidence that the effect of context on scalar inferencing emerged only at long lag times. As illustrated in Figure 2, the effect of context (at “the rest”) remains the same regardless of the lag time.

## Discussion

The present results are consistent with previous studies in showing that a scalar inference is more likely to be made in an upper-bound than a lower-bound context, as “the rest” was read faster in the former context. However, contrary to BKW, the quantifier “some of” and the following words were not read more slowly in the upper-bound context. Since previous studies (BKW, [5], [22]) assumed that processing cost associated with inferencing would result in longer reading times for quantifiers in an inference-triggering (e.g., upper-bound) compared to a non-inference-triggering (lower-bound) context, the lack of a slowdown in the present study challenges the notion that the realization of the inference was effortful. The present findings suggest that the reading time slowdown observed by BKW was due to properties of their stimuli other than the pragmatic manipulation. We also found the inference occurring earlier than in H&S, failing to replicate their finding that the effect of context on “the rest” only emerged at a long lag after the quantifier; this suggests that their result may be due to other structural properties of the stimuli and not to the speed of inference (for instance, H&S’s long-lag conditions included adverbial phrases after the quantifier, while the short-lag condition did not). Overall, then, the present study did not provide direct evidence that the realization of a scalar inference was costly in the context tested.

An anonymous reviewer suggested that the results may be consistent with context-driven models if one assumes that comprehenders do not realize the inference until they encounter language referring to the complement set (“the rest”). Under such an account, the lack of reading time difference observed at the quantifier would be due to the fact that the inference was not realized in either condition (note that this is different than the account made in previous self-paced reading studies, which assumed that the inference would be realized at the quantifier position in upper-bound context and the extra computations necessary for realizing the inference would result in increased reading times). The reading time slowdown at “the rest” in the lower-bound context, then, would be due to participants’ extra effort required to integrate the complement set with the preceding context. We note that such an account would still not provide direct evidence of processing cost associated with scalar inferencing–rather, it would suggest evidence of avoidance of inferencing entirely. It does not seem likely that inferences are only realized when a complement set is mentioned, given that inferences seem to be derived in sentences with no complement set (e.g., example (1b) in this article) and there is substantial experimental evidence for rapid and implicit sensitivity to inference-based meanings even in the absence of complement sets ([23], [24], among others). Moreover, anticipatory eye movements reflecting the realization of inferences have been observed following quantifiers, but before the mention of a contrast set or any subsequent referring expression [8], [25].

The present results thus raise questions for context-based models. While numerous recent studies have suggested that inferences are realized at a delay except in special contexts (e.g., [4], [10], H&S), the traditional explanation for that finding is that inferencing is effortful and thus the parser avoids inferencing until after it can evaluate whether the extra effort is worthwhile, or at least until after the core semantic meaning of the scalar term has already been realized. If inferencing is not effortful, then a new explanation for the delay would be needed (see [20], for several alternative accounts). Alternatively, inferencing may be effortful but reading times may not be sensitive to this effort–in addition to the present study, H&S (self-paced reading) and Lewis & Phillips (unpublished eye-tracking data) have failed to find processing effort for the quantifier in inference-supporting contexts. If that is the case, future studies must use other methods to test for different instantiations of processing costs. This may be accomplished both using direct measures of processing cost (such as, possibly, event-related potentials) and via indirect means (such as testing whether individual differences in various cognitive abilities predict the extent to which individuals inference online). We believe both these routes can make important contributions to examining context-driven accounts of inferencing.

It nevertheless seems unlikely that the lack of a context effect at the quantifier in the present study is due just to reading times not being sensitive enough. A recent experiment [22] did find evidence for a reading time slowdown at the quantifier position in a similar study, and the effect could not have been due to repeated lexical items as it was in BKW. This provides evidence that self-paced reading times can indeed be sensitive to processing effort in scalar inferencing, making it less likely that the null effect observed in the present study was simply due to the the dependent measure used. As [22] used a different context manipulation than this and previous studies (they manipulated the speaker’s epistemic state, whereas the present study manipulated information-structural constraints), the comparison between their findings and the present results raises the possibility that inferencing may be costly in certain contexts and not in others. Such a conclusion would be consistent with the constraint-based account of inferencing [9] discussed below.

The present study also raises questions for default accounts–specifically, while a default model could account for the present findings (by assuming that the inference was effortlessly realized at “some of” and then cancelled in the lower-bound context before “the rest”), default models owe an account of the nature of inference cancellation and the processes that underlie it. [6] describes two algorithms for determining whether a default inference will be cancelled. The first involves checking whether an inference is consistent with the previous context or higher-ranked information (e.g., in example (2b), the inference “not all of the students are hardworking” is inconsistent with the explicit entailment “all of the students are [hardworking]”; [5] also make reference to additional epistemic factors in the context which could cause an inference to be cancelled or not realized, such as if a speaker is known to be non-cooperative). The lower-bounded contexts in the present study would not trigger inference cancellation from this mechanism, since the inference does not conflict with information in the sentence or prevent the comprehender from completing the task (i.e., the question of “whether *any* of John’s relatives are staying in his apartment” is answered even if the answer is “*some but not all* of them are”). Therefore, the fact that the inference was cancelled in lower-bound contexts before “the rest” (as evidenced by slower reading times to “the rest” in that context) would have to be explained through the second cancellation mechanism described by [6], whereby inferences that are irrelevant to the goal of the conversation are discarded. However, BKW (see also [5]) assume that inference cancellation should involve extra effort, and some experimental evidence also suggests that it does [15], [24]. If the processor avoids unnecessary effort, it is unclear why it would make the effort to cancel inferences that do not interfere with the comprehension of the utterance. As suggested by [6], the default model is lacking a full account of what about this particular context would cause inference cancellation, and the nature of the process through which inferences are cancelled; the results of the present study highlight the need for such an account if the default model is to explain how meaning is realized online in the contexts tested in this experiment.

The present results may be amenable to the constraint-based account proposed by [9]. Under this account, scalar inferencing is a result of rapid integration of multiple constraints, which may facilitate or inhibit the inference. Unlike traditional context-driven accounts, this account may predict that inferencing is both context-sensitive and potentially rapid and effortless. If numerous constraints strongly facilitate the inference, then realizing the inference may not require great effort; on the other hand, if constraints discourage the comprehender from making the inference, it may not be realized at all. Such a model would be able to account for seemingly effortless inferencing in contexts like the upper-bound context of the present study. This is different from traditional context-driven models, which assume that inferencing is always costly and therefore that when it does happen it will be late and effortful. Further study would be useful to investigate the predictions of a constraint-based account for this type of paradigm.

In conclusion, the present study raises questions for both default and context-driven accounts of inferencing, and suggests that alternative accounts or reformulations of these accounts may be worth considering. The results also challenge the field to seek evidence for processing costs in new ways. Both of these endeavors have the potential to improve our understanding of how comprehenders compose the meaning of utterances in real-time.

## Supporting Information

File S1 **Stimuli.** List of stimuli used in the experiments. Stimuli for target sentences are shown on the first tab; stimuli for filler sentences are shown on the second tab.
(XLS)

File S2 **Reading time and accuracy data.** The data are in the form of a zipped file which includes an R data file that can be loaded in R (http://www.r-project.org/). The reading time data and accuracy data are stored as separate objects within that file. After loading the data in R, a description of the objects can be viewed by typing cat(Explanation) at the command line.
(ZIP)

Appendix S1 **Power analysis.** A document describing the power analysis that was conducted.
(PDF)
