# Supplementary material for: The Realization of Scalar Inferences: Context Sensitivity without Processing Cost
Source: PLoS One. 2013 May 16;8(5):e63943. doi: 10.1371/journal.pone.0063943 (PMC3655969; doi:10.1371/journal.pone.0063943)
Supplement: Appendix S1 — Power analysis. A document describing the power analysis that was conducted. (PDF) [file pone.0063943.s003.pdf]

The power analysis was conducted using Monte Carlo simulation, following the method described by [1]. In this method, the researcher simulates a population in which the effect under question (in this case, the slowdown at the quantifier observed in [2]), and generates numerous samples (at least 1000) that have comparable size and variance structure as the actual data. The proportion of times the simulated effect is detected in the random samples is the statistical power to detect that effect from a given sample size.

For our analysis, the population parameters (estimates for fixed effects, and standard deviations for random and fixed effects) were estimated based on the model of the actual data collected, except that the parameter for the effect of Boundedness at the quantifier in *some of* sentences was instead set to the size of the slowdown observed in (log(1027) – log(927) = 0.1024436). Using normal distributions with these population parameters, 5000 data sets with the same number of participants and items as in the present study were simulated. For each data set, a linear mixed effects model was calculated using the same fixed and random parameters as in the model calculated on the actual data. The $t$ values for the critical effect (the effect of Boundedness at the quantifier in *some of* sentences) were stored, and across all simulations the proportion of models in which the $t$ value for this effect exceeded 2 was calculated. This proportion is the power.

## References


1 Gelman A, Hill J (2006) Data analysis using regression and multilevel/hierarchical models. Cambridge: Cambridge University Press. 648 p.

2 Breheny R, Katsos N, Williams J (2006) Are generalized scalar implicatures generated by default? An on-line investigation into the role of context in generating pragmatic inferences. Cognition 100: 434-463.
